# S205. TRANSCRANIAL DIRECT CURRENT STIMULATION FOR SEVERE, PERSISTENT, TREATMENT-REFRACTORY AUDITORY HALLUCINATIONS IN SCHIZOPHRENIA

**DOI:** 10.1093/schbul/sby018.992

**Published:** 2018-04-01

**Authors:** Parth Goyal, Lakhan Kataria, Chittaranjan Andrade, Priyal Desai

**Affiliations:** 1 SBKS MI&RC; 2 NIMHANS, Bengaluru

## Abstract

**Background:**

Up to 25% of schizophrenia patients continue to experience distressing auditory hallucinations despite best efforts at treatment with antipsychotic drugs. Transcranial direct current stimulation (tDCS) has been suggested to rapidly attenuate such persistent hallucinations.

**Methods:**

We treated 23 schizophrenia patients with persistent, antipsychotic-refractory auditory hallucinations using tDCS in a single-group, open-label design. tDCS was administered at 2 mA current intensity for 20 min, twice-daily and 4 h apart, across 5 consecutive days; the anode was placed over the the left dorsolateral prefrontal cortex and the cathode over the left temporoparietal junction. Ongoing antipsychotic medications were continued unchanged. Patients were assessed using the Auditory Hallucinations Rating Scale (AHRS) at treatment endpoint and at 1- and 3-month follow up. Response was defined as 50% or greater attenuation in AHRS scores.

**Results:**

All patients completed the study. tDCS resulted in substantial improvement. Mean (standard deviation) AHRS scores dropped from 29.0(8.3) at baseline to 4.4(5.6) at treatment endpoint; these values were 9.3(9.3) and 7.8(8.4) at 1- and 3-months follow up. The response rate was 91.3%, 69.6%, and 82.6% at the 3 posttreatment assessment points, respectively. Complete remission of hallucinations (AHRS=0) was observed in 61%, 44%, and 44% at the 3 posttreatment assessment points. tDCS was very well tolerated and adverse effects were minimal.

**Discussion:**

tDCS is effective and well tolerated in schizophrenia patients with persistent, antipsychotic-refractory auditory hallucinations. In most patients, the benefits last for up to 3 months or longer.

